# Cooperation and the Fate of Microbial Societies

**DOI:** 10.1371/journal.pbio.1001549

**Published:** 2013-04-30

**Authors:** Benjamin Allen, Martin A. Nowak

**Affiliations:** 1 Department of Mathematics, Emmanuel College, Boston, Massachusetts, United States of America; 2 Program for Evolutionary Dynamics, Harvard University, Cambridge, Massachusetts, United States of America; 3 Department of Mathematics, Department of Organismic and Evolutionary Biology, Harvard University, Cambridge, Massachusetts, United States of America

## Abstract

Microorganisms have been cooperating with each other for billions of years: by sharing resources, communicating with each other, and joining together to form biofilms and other large structures. These cooperative behaviors benefit the colony as a whole; however, they may be costly to the individuals performing them. This raises the question of how such cooperation can arise from natural selection. Mathematical modeling is one important avenue for exploring this question. Evolutionary experiments are another, providing us with an opportunity to see evolutionary dynamics in action and allowing us to test predictions arising from mathematical models. A new study in this issue of *PLOS Biology* investigates the evolution of a cooperative resource-sharing behavior in yeast. Examining the competition between cooperating and “cheating” strains of yeast, the authors find that, depending on the initial mix of strains, this yeast society either evolves toward a stable coexistence or collapses for lack of cooperation. Using a simple mathematical model, they show how these dynamics arise from *eco-evolutionary feedback*, where changes in the frequencies of strains are coupled with changes in population size. This study and others illustrate the combined power of modeling and experiment to elucidate the origins of cooperation and other fundamental questions in evolutionary biology.

How much cooperation does it take to maintain a society? Many biological populations, from microbes to insects to humans, depend on the cooperation of their members in order to access resources, raise offspring, and avoid danger. Yet in any cooperative activity, there is the risk of “cheaters,” who benefit from the generosity of others while making no contribution of their own. Consider, for example, the layabout in a communal household who refuses to cook or clean dishes. If this cheating behavior spreads through the population, the society as a whole may collapse.

Evolutionary biologists since Darwin have been fascinated by how populations can overcome this dilemma. Studying this question can be challenging. While the products of evolution are evident in the natural world, the process that produced them is mostly hidden from view. As a consequence, direct observation of the evolution of cooperation in action is often limited.

Much of our current understanding of this conundrum arises from mathematical modeling. Ever since the birth of population genetics about a century ago, it has been recognized that the theory of evolution can be set upon exact mathematical foundations. This approach has flourished ever since, and especially in the last few decades. The theory of choice to study social phenomena is evolutionary game theory [1]–[5], in which behaviors that affect others are represented as strategies. Simple mathematical models describe the dynamics of these strategies under mutation and selection, depending on the population structure [6]–[12]. Applied to the problem of cooperation, these models show that if a cooperating individual receives some of the benefit of his or her own labors—as in Snowdrift games or some nonlinear public goods games—then evolutionary dynamics may lead to an equilibrium in which cooperators and cheaters coexist [1],[13]. On the other hand, if benefits accrue only to others—as in Prisoners' Dilemma games—then cooperation is expected to disappear unless some mechanism is present to support it [14].

Recently, experiments with microbes have afforded us an unprecedented opportunity to observe evolution in action [15]–[20]. Bacteria, yeast, and other single-celled organisms divide rapidly enough that evolutionary change—the arrival and fixation of beneficial mutations—can be observed in the laboratory. Moreover, the experimenter is able to control the population size, environmental conditions, and other variables, and can therefore test hypotheses regarding how the course of evolution depends on these variables. Experimenters can also preserve specimens of the population from all phases of its evolution as a “living record” of genotypic and phenotypic change. In short, experiments with microbes are a powerful tool for testing evolutionary hypotheses.

Microorganism experiments hold particular promise for shedding light on how cooperative behaviors emerge from evolution [21]–[26]. Microbial species cooperate in a variety of ways: They form biofilms, produce iron-scavenging agents, produce chemicals to resist antibiotics, and form fruiting bodies when local resources are depleted. By mixing wild-type strains that display a particular cooperative behavior with “cheater” mutants that do not, researchers can test hypotheses about what conditions favor wild-type “cooperators” over cheaters.

In one such experiment, Gore et al. [26] studied a social dilemma in the yeast *Saccharomyces cerevisiae*. The preferred nutrient sources for this yeast are the simple sugars glucose and fructose; however, it can subsist on the compound sugar sucrose by producing the enzyme invertase, which breaks down sucrose into glucose and fructose. A crucial point is that, since this reaction occurs near the cell wall, only about 1% of these simple sugars are captured by the cell in which they are produced. The remaining 99% diffuse away and are available to other cells. Thus producing invertase is a cooperative behavior, with the bulk of the benefit going to cells other than the producer. Moreover, this cooperation is costly, in that the production of invertase carries a metabolic cost to the producer. To study the evolution of this behavior, Gore et al. created cheater strains that do not produce invertase, and thereby avoid the associated cost. Letting these strains compete with each other, they found that, in most cases, cooperator and cheater strains converged to an equilibrium in which both strains coexisted—a result consistent with theoretical predictions regarding Snowdrift games and nonlinear public goods games [1],[13].

Much theoretical work on the evolution of cooperation and other traits has assumed, for the sake of simplicity, that the population size remains roughly constant while the strains in question are competing. However, it is entirely possible that *population dynamics*—changes in population size—may occur on the same timescale as *evolutionary dynamics—*changes in the frequencies of competing types. In this case, these two dynamical processes may affect one another, a phenomenon known as *eco-evolutionary feedback* [27]–[30]. Mathematical modeling has shown that eco-evolutionary feedback may lead to a variety of complex dynamical behaviors, including multiple equilibria, cycling, chaos, and Turing patterns [28],[30]–[33].

In this issue of *PLOS Biology*, Sanchez and Gore [34] have—for the first time, to our knowledge—empirically demonstrated eco-evolutionary feedback in the evolution of cooperation. Using the yeast system described above, the authors studied the coupled dynamics of the population density and the proportion of cooperator types within the population. The mechanism for eco-evolutionary feedback in this system is intuitive: the growth of the population as a whole depends on the concentration of simple sugars, which in turn depends on the density of cooperators. If there are insufficient cooperators, the overall population density declines. With low population density, cooperators have an advantage due to the simple sugars they manage to retain for themselves. At this point, cooperators increase in frequency, and the concentration of simple sugars increases, leading to overall population growth. But once this happens, cheaters proliferate faster than cooperators due to their lower metabolic costs. This in turn depresses the frequency of cooperators, and the cycle repeats itself. We would therefore expect to see cycling or spiraling behavior in the eco-evolutionary dynamics of these types, consistent with theoretical predictions [32],[33].

In their experiment, Sanchez and Gore observed not only spiraling, but also *bistability—*the presence of two equilibria to which the system might converge, depending on the initial conditions [35]. If the initial population density and/or the initial proportion of cooperators is too low, not enough simple sugars are produced and the population collapses. On the other hand, if there are sufficiently many cooperators in the initial population, the population converges in spiraling fashion to an equilibrium in which population density is high and cooperators and cheaters coexist (Figure 1). To complement their experiment, the authors developed a simple Lotka-Volterra–type model describing the interdependent growth of the competing strains. This model reproduces the observed eco-evolutionary dynamics with remarkable fidelity, given its simplicity.

**Figure 1:**
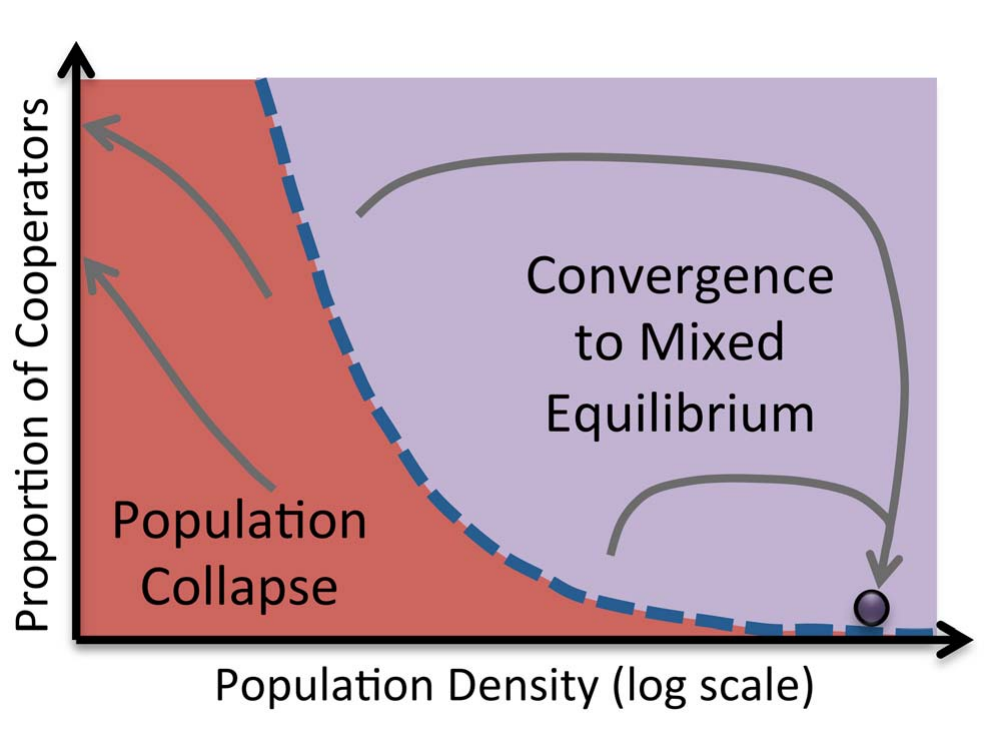
Dynamics of eco-evolutionary feedback in cooperator and cheater strains of the yeast *S. cerevisiae* , as observed in the experiment of Sanchez and Gore. There are two basins of attraction, with a different outcome expected from each. If there are too few cooperators to start, not enough simple sugars are produced and the population collapses. On the other hand, if the initial number of cooperators is sufficient, the system converges in spiraling fashion to an equilibrium in which cooperators and cheaters coexist.

Interestingly, the proportion of cooperators in the coexistence equilibrium is low—less than 10%—but is nonetheless sufficient to maintain the viability of the population. Does the predominance of cheaters in this equilibrium hurt the population as a whole? The authors found that the overall density and productivity of the population in the coexistence equilibrium is not much less than what cooperators would achieve in the absence of cheaters. However, the predominance of cheaters does impact the population's resilience to an ecological shock—in this case, rapid and significant dilution of the population. Cooperators in monomorphic equilibrium survive this shock, but populations in mixed equilibrium between cooperators and cheaters do not. In short, mixed populations are comparably productive to, but significantly less resilient than, cooperator-only populations.

The study of Sanchez and Gore illustrates the synergistic power of theory and experiment when carefully combined. The opportunities for further such combinations are immense. Population genetics and evolutionary game theory have provided us with a wealth of testable hypotheses about evolution, and we now have the experimental technology to test them. Some of the most interesting hypotheses regard the effect of spatial structure on the evolution of cooperation. Well-known results in evolutionary game theory show that spatial structure can promote cooperation [6],[36]–[39], though this effect depends strongly on the details of spatial reproduction and replacement [40]. Thus far, experimental studies have addressed this question only indirectly, with reduced pathogen virulence representing an indirect form of cooperation [41], or with group subdivision standing in for spatial structure [22],[23]. The effects of spatial structure on the evolution of cooperation in microbial colonies remains an important open question.

At the same time, we must also allow experimental results to inform the development of new mathematical models. The field of social bacterial evolution requires well-defined, simple models that describe how populations of bacteria change over time, taking into account the reproductive events, social interactions, and population structures particular to these populations. This approach ultimately brings together the methods of population genetics, evolutionary game theory, ecology, and experimental microbiology.

## References

[1] Maynard Smith J, Price GR (1973) The logic of animal conflict. Nature 246: 15–18.

[2] Nowak MA, Sigmund K (2004) Evolutionary dynamics of biological games. Science 303: 793–799. 14764867 10.1126/science.1093411

[3] Weibull JW (1997) Evolutionary game theory. Cambridge (Massachusetts): MIT Press.

[4] Cressman R (1992) The stability concept of evolutionary game theory: a dynamic approach. Berlin: Springer-Verlag.

[5] Hofbauer J, Sigmund K (2003) Evolutionary game dynamics. Bull New Ser Am Math Soc 40: 479–519.

[6] Durrett R, Levin S (1994) The importance of being discrete (and spatial). Theor Popul Biol 46: 363–394.

[7] Hassell MP, Comins HN, May RM (1994) Species coexistence and self-organizing spatial dynamics. Nature 370: 290–292.

[8] Tilman D, Kareiva P (1997) Spatial ecology: the role of space in population dynamics and interspecific interactions. Princeton (New Jersey): Princeton University Press.

[9] Szabó G, Fáth G (2007) Evolutionary games on graphs. Phys Rep 446: 97–216.

[10] Nowak MA, Tarnita CE, Wilson EO (2010) The evolution of eusociality. Nature 466: 1057–1062. 20740005 10.1038/nature09205 PMC3279739

[11] Nowak MA, Tarnita CE, Antal T (2010) Evolutionary dynamics in structured populations. Philos Trans R Soc Lond B Biol Sci 365: 19–30. 20008382 10.1098/rstb.2009.0215 PMC2842709

[12] Allen B, Tarnita CE (2012) Measures of success in a class of evolutionary models with fixed population size and structure. J Math Biol E-pub ahead of print. 10.1007/s00285-012-0622-x 23179131

[13] Archetti M, Scheuring I (2012) Review: game theory of public goods in one-shot social dilemmas without assortment. J Theor Biol 299: 9–20. 21723299 10.1016/j.jtbi.2011.06.018

[14] Nowak MA (2006) Five rules for the evolution of cooperation. Science 314: 1560–1563. 17158317 10.1126/science.1133755 PMC3279745

[15] Barrick JE, Yu DS, Yoon SH, Jeong H, Oh TK, et al (2009) Genome evolution and adaptation in a long-term experiment with Escherichia coli. Nature 461: 1243–1247. 19838166 10.1038/nature08480

[16] Elena SF, Lenski RE (2003) Evolution experiments with microorganisms: the dynamics and genetic bases of adaptation. Nat Rev Genet 4: 457–469. 12776215 10.1038/nrg1088

[17] Kerr B, Riley MA, Feldman MW, Bohannan BJM (2002) Local dispersal promotes biodiversity in a real-life game of rock-paper-scissors. Nature 418: 171–174. 12110887 10.1038/nature00823

[18] Segre D, DeLuna A, Church GM, Kishony R (2004) Modular epistasis in yeast metabolism. Nat Genet 37: 77–83. 15592468 10.1038/ng1489

[19] Weinreich DM, Delaney NF, DePristo MA, Hartl DL (2006) Darwinian evolution can follow only very few mutational paths to fitter proteins. Science 312: 111–114. 16601193 10.1126/science.1123539

[20] Hallatschek O, Hersen P, Ramanathan S, Nelson DR (2007) Genetic drift at expanding frontiers promotes gene segregation. Proc Natl Acad Sci U S A 104: 19926–19930. 18056799 10.1073/pnas.0710150104 PMC2148399

[21] Branda SS, González-Pastor JE, Ben-Yehuda S, Losick R, Kolter R (2001) Fruiting body formation by *Bacillus subtilis* . Proc Natl Acad Sci U S A 98: 11621–11626. 11572999 10.1073/pnas.191384198 PMC58779

[22] Chuang JS, Rivoire O, Leibler S (2009) Simpson's paradox in a synthetic microbial system. Science 323: 272–275. 19131632 10.1126/science.1166739

[23] Griffin AS, West SA, Buckling A (2004) Cooperation and competition in pathogenic bacteria. Nature 430: 1024–1027. 15329720 10.1038/nature02744

[24] Rainey PB, Rainey K (2003) Evolution of cooperation and conflict in experimental bacterial populations. Nature 425: 72–74. 12955142 10.1038/nature01906

[25] Velicer GJ, Kroos L, Lenski RE (2000) Developmental cheating in the social bacterium *Myxococcus xanthus* . Nature 404: 598–601. 10766241 10.1038/35007066

[26] Gore J, Youk H, van Oudenaarden A (2009) Snowdrift game dynamics and facultative cheating in yeast. Nature 459: 253–256. 19349960 10.1038/nature07921 PMC2888597

[27] Hanski IA (2011) Eco-evolutionary spatial dynamics in the Glanville fritillary butterfly. Proc Natl Acad Sci U S A 108: 14397–14404. 21788506 10.1073/pnas.1110020108 PMC3167532

[28] Post DM, Palkovacs EP (2009) Eco-evolutionary feedbacks in community and ecosystem ecology: interactions between the ecological theatre and the evolutionary play. Philos Trans R Soc Lond B Biol Sci 364: 1629–1640. 19414476 10.1098/rstb.2009.0012 PMC2690506

[29] Schoener TW (2011) The newest synthesis: understanding the interplay of evolutionary and ecological dynamics. Science 331: 426–429. 21273479 10.1126/science.1193954

[30] Pelletier F, Garant D, Hendry A (2009) Eco-evolutionary dynamics. Philos Trans R Soc Lond B Biol Sci 364: 1483–1489. 19414463 10.1098/rstb.2009.0027 PMC2690510

[31] Wakano JY, Nowak MA, Hauert C (2009) Spatial dynamics of ecological public goods. Proc Natl Acad Sci U S A 106: 7910–7914. 19416839 10.1073/pnas.0812644106 PMC2683138

[32] Hauert C, Holmes M, Doebeli M (2006) Evolutionary games and population dynamics: maintenance of cooperation in public goods games. Philos Trans R Soc Lond B Biol Sci 273: 2565–2571. 10.1098/rspb.2006.3600 PMC1634915 16959650

[33] Hauert C, Wakano JY, Doebeli M (2008) Ecological public goods games: cooperation and bifurcation. Theor Popul Biol 73: 257–263. 18221761 10.1016/j.tpb.2007.11.007 PMC2276362

[34] Sanchez A, Gore J (2013) Feedback between population and evolutionary dynamics determines the fate of social microbial populations. PLoS Biol 11: e1001547 doi:10.1371/journal.pbio.1001547. 23637571 10.1371/journal.pbio.1001547 PMC3640081

[35] Dai L, Vorselen D, Korolev KS, Gore J (2012) Generic indicators for loss of resilience before a tipping point leading to population collapse. Science 336: 1175–1177. 22654061 10.1126/science.1219805

[36] Nowak MA, May RM (1992) Evolutionary games and spatial chaos. Nature 359: 826–829.

[37] Van Baalen M, Rand DA (1998) The unit of selection in viscous populations and the evolution of altruism. J Theor Biol 193: 631–648. 9750181 10.1006/jtbi.1998.0730

[38] Ohtsuki H, Hauert C, Lieberman E, Nowak MA (2006) A simple rule for the evolution of cooperation on graphs and social networks. Nature 441: 502–505. 16724065 10.1038/nature04605 PMC2430087

[39] Taylor PD, Day T, Wild G (2007) Evolution of cooperation in a finite homogeneous graph. Nature 447: 469–472. 17522682 10.1038/nature05784

[40] Hauert C, Doebeli M (2004) Spatial structure often inhibits the evolution of cooperation in the snowdrift game. Nature 428: 643–646. 15074318 10.1038/nature02360

[41] Kerr B, Neuhauser C, Bohannan BJM, Dean AM (2006) Local migration promotes competitive restraint in a host–pathogen ‘tragedy of the commons’. Nature 442: 75–78. 16823452 10.1038/nature04864

